# The Eurasian invasion: phylogenomic data reveal multiple Southeast Asian origins for Indian Dragon Lizards

**DOI:** 10.1186/s12862-016-0611-6

**Published:** 2016-02-19

**Authors:** Jesse L. Grismer, James A. Schulte, Alana Alexander, Philipp Wagner, Scott L. Travers, Matt D. Buehler, Luke J. Welton, Rafe M. Brown

**Affiliations:** Department of Ecology and Evolutionary Biology and Biodiversity Institute, University of Kansas, Dyche Hall, 1345 Jayhawk Blvd., Lawrence, KS 66045-7561 USA; Department of Biology, Clarkson University, 8 Clarkson Avenue, Postdam, NY 13699 USA; Zoologisches Forschungsmuseum Alexander Koenig Adenauerallee 160, D-53113 Bonn, Germany

**Keywords:** Agamidae, Draconinae, Eocene, Eurasia, India, Faunal exchanges, Landbridges

## Abstract

**Background:**

The Indian Tectonic Plate split from Gondwanaland approximately 120 MYA and set the Indian subcontinent on a ~ 100 million year collision course with Eurasia. Many phylogenetic studies have demonstrated the Indian subcontinent brought with it an array of endemic faunas that evolved *in situ* during its journey, suggesting this isolated subcontinent served as a source of biodiversity subsequent to its collision with Eurasia. However, recent molecular studies suggest that Eurasia may have served as the faunal source for some of India’s biodiversity, colonizing the subcontinent through land bridges between India and Eurasia during the early to middle Eocene (~35–40 MYA). In this study we investigate whether the Draconinae subfamily of the lizard family Agamidae is of Eurasian or Indian origin, using a multi locus Sanger dataset and a novel dataset of 4536 ultraconserved nuclear element loci.

**Results:**

Results from our phylogenetic and biogeographic analyses revealed support for two independent colonizations of India from Eurasian ancestors during the early to late Eocene prior to the subcontinent’s hard collision with Eurasia.

**Conclusion:**

These results are consistent with other faunal groups and new geologic models that suggest ephemeral Eocene land bridges may have allowed for dispersal and exchange of floras and faunas between India and Eurasia during the Eocene.

**Electronic supplementary material:**

The online version of this article (doi:10.1186/s12862-016-0611-6) contains supplementary material, which is available to authorized users.

## Background

The collision of the Indian subcontinent (ISC) into Eurasia caused the formation of some of the world’s most iconic deserts and mountain ranges, dramatically changing Asian climates, while simultaneously sculpting its biodiversity. Much interest has centered on investigating the evolutionary and geological processes that have influenced the origins and diversification of the ISC’s unique biotas ([1]; and references therein). Phylogenetic studies of birds, dipterocarp trees, terrestrial gastropods, crabs, freshwater fish, and certain groups of amphibians, suggests these lineages originated on the ISC and were a source of biodiversity for regions of Asia and areas as far west as Africa after the Indian Plate split off from Gondwanaland [2–7]. However, a suite of phylogenetic studies across a variety of other taxa suggest an alternative biogeographic hypothesis postulating Eurasia as the ancestral source of diversity for the ISC. In these groups Asian lineages dispersed to, and successfully colonized, the subcontinent before its hard collision with Eurasia 25–30 MYA [8–12].

The previous lack of geologic models describing the fine scale events of the final 50 million years of the ISC’s collision, left researchers with no mechanistic explanation for the striking differences between these two “ISC faunal origin” hypotheses. Fortunately, newer models are available that take into account continental connections between the approaching ISC and areas of mainland Asia prior to the ISC’s collision with Eurasia [13–15]. Acton [13] and Ali and Aitchison [15] hypothesized that between 34–55 MYA (middle Eocene-late Eocene), India was connected to Eurasia via land-bridges with Sumatra, and then along what is now the Thai-Malay Peninsula and Burma (which would have been one land mass during this time). Two recent studies have recovered phylogenetic support for these Eocene land bridges and hypothesized that these pre-collision continental connections would have allowed for faunal exchanges between the ISC and Eurasia as the ISC continued northward [7, 16]. We present data from a diverse radiation of Indian and Southeast Asian lizards that provide an additional model system, with largershould be large not "larger" amounts of generic diversity of Indian lineages and Asian lineages, to test for phylogenetic support for these Eocene land bridges, which we refer to as the Eocene Exchange Hypothesis (EEH).

**Fig. 1:**
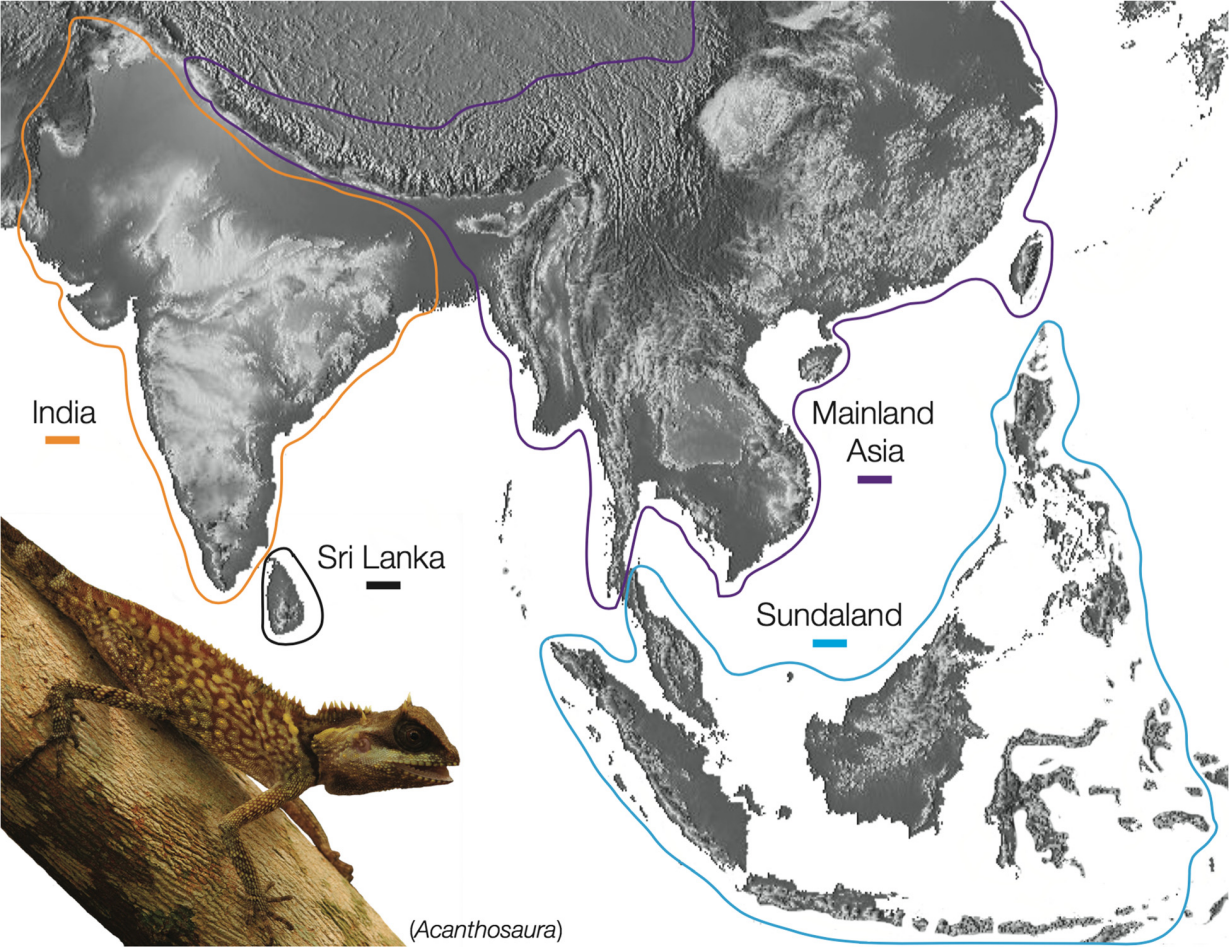
Map showing the distribution of Draconinae and the four biogeographic area (differently-colored borders) used in ancestral range reconstructions

The Draconinae is a subfamily within the lizard family Agamidae that contains 27 genera and 199 species [17] comprising approximately 50 % of total Agamid diversity. Members of the Draconinae collectively range throughout mainland Asia (Indochina), Sundaland, India, and Sri Lanka (Fig. 1). Draconinae lizards are diurnal omnivores exhibiting a range of arboreal and terrestrial life styles and are some of the dominant members of diurnal lizard communities throughout South and Southeast Asia [18, 19]. To date, only two studies have investigated the phylogenetic relationships within the Draconinae. However, both were part of broader systematic studies on the entire Agamidae family [20, 21]. Moody’s [20] dissertation included 60 extant taxa, was based on 122 morphological characters, and included data from 18 fossils. This work was the first study to hypothesize a Eurasian origin for the Indian draconine lineages. Macey et al. [21] was the first study to provide a molecular phylogeny for the Agamidae (including Draconinae), and included an analysis of 72 taxa and one mitochondrial gene. This analysis demonstrated that mainland Asian agamids were paraphyletic with respect to Indian and Sri Lankan lineages. However, multiple deeper nodes within the Draconinae were characterized by poor support, resulting in ambiguous relationships [21]. The authors then used a series of parsimony methods to suggest that these problematic areas of the draconine phylogeny, along with a lack of biogeographic signal, were likely due to an Indian-Asian faunal exchange just after the hard collision, 20–25 MYA. Subsequent reviews of Indian-Eurasian collision regarded the biogeographical interpretations of Macey et al. [21] with skepticism due to the poorly supported relationships within the Draconinae ([22]; and references therein).

Since Moody [20] and Macey et al. [21], new Draconinae genera have been discovered, and previously unsampled rare genera have been collected, providing additional genetic material for reanalysis of draconine relationships. The lower per-base cost of next-generation sequencing has also led to the development of genomic methods extending the number of genetic markers that have limited the phylogenetic resolution in previous studies. Here, we generate a genomic data set of 4536 nuclear loci derived from ultraconserved elements (UCEs), along with traditional Sanger sequencing data, to resolve the problematic relationships within the Draconinae reported by Macey et al. [21]. With the addition of new taxa, and genomic sequence-capture data, analyzed in combination with newly developed geological models, we are poised to reinterpret the biogeographic origins of Indian and Southeast Asian draconine lineages. Specifically, we tested (1) Moody’s [20] pre-collision hypothesis versus Macey et al. [21] post-collision hypothesis for the origins of Indian lineages; and (2) suggest that a conclusion in favor of Moody’s [20] pre-collision hypothesis would show phylogenetic support for the Eocene land bridge connections proposed by Acton [13] and Atchison et al. [14]. We term this the Eocene Exchange Hypothesis (EEH).

## Methods

### DNA extraction, Sanger mitochondrial and nuclear DNA sequence data collection

Genomic DNA was extracted from muscle or liver tissue samples on loan form La Sierra University, Villanova University, the California Academy of Sciences, the Zoologisches Forschungsmuseum Alexander Koenig, and the Chicago Field Museum. Extractions were preformed using a DNeasy tissue kit (Qiagen, Inc.) and sequenced for the mitochondrial and nuclear genes, ND2 (primers from [21]) and RAG-1 (primers from [23]), respectively, using standard PCR and Sanger sequencing protocols. We edited the sequences and aligned them within Geneious Pro 5.0.4 (http://www.geneious.com, [24]) and these new sequence data were combined with existing data from [21] and [23] (Additional file 1: Table S1). In total, the dataset included 17 of the 26 draconine genera, including all but two of the Indian genera (*Psammophilus* and *Coryphophylax*). *Hyrdosaurus* and *Physignathus* were not included as their phylogenetic affinities are with other agamid lineages outside of the Draconinae [21]. At least three species (or individuals if the genus was monotypic) per genus were sampled, for a total of 44 individuals. ND2 and RAG-1 were selected as they are the most frequently sequenced markers across acrodont lizards and therefore provide maximum taxonomic coverage. We used these markers to preliminarily place new genera in a phylogenetic context, and as a guide tree in our selection of genera for UCE development to resolve problematic relationships. No experimental research was carried out on these animals in this study.

#### Ultraconserved elements (UCE) data collection

**Fig. 2:**
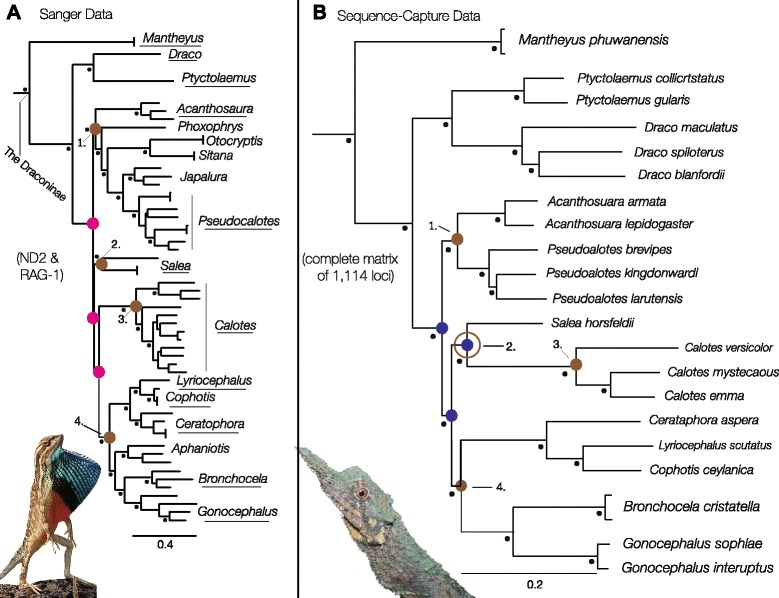
**a** Bayesian analysis (in MrBayes) of ND2 and RAG-1 data, with black dots denoting nodes with posterior probabilities above 0.95. Brown nodes indicate four well-supported species groups (1–4; see text for details) and pink nodes identify poorly supported relationships among these species groups. Underlined taxon names are genera selected for UCE enrichment. **b** Multi-species coalescent (“species tree”) from the species tree estimation using average coalescence times STEAC analysis, using the complete matrix of 1114 UCE loci. Black dots denote nodes with 100 bootstrap support. Brown nodes indicate the four species groups (Group 2 = brown circle; see text for discussion). Blue nodes identify problematic nodes recovered in Likelihood analysis of the Sanger dataset, resolved with sequence-capture data

To resolve the problematic areas in the phylogeny from the Sanger data (pink nodes: Fig. 2a), we selected 24 individuals representing 12 genera (underlined taxon names in Fig. 2) from across four species groups (brown nodes: Fig. 2a) for ultaconserved element (UCE) enrichment. Sequence-capture data collection followed a modification of the approach outlined by Faircloth et al. [25]. Briefly, we fragmented genomic DNA with a Covaris S220 ultrasonicator (Covaris, Inc.), and prepared Illumina libraries using KAPA library preparation kits (Kapa Biosystems) and custom sequence tags unique to each sample [26]. Libraries were pooled into groups of 8 taxa and enriched for 5060 UCE loci (5472 probes). We amplified enriched pools with a limited-cycle PCR (18 cycles) and sequenced final libraries on a partial Ilumina HiSeq 2000 lane. Reads were quality filtered using the Illumiprocessor [27] wrapper for Trimmomatric [28], and assembled into contigs using Trinity [29]. Where alternate alleles differing by less than 5 % sequence divergence (or two nucleotide positions, whatever was greater) were present in a sample for any given UCE locus, Trinity retained the allele supported by the largest number of reads. We used PHYLUCE v. 1.4 (Faircloth et al. [25, 30]) to match contigs to UCE loci and generated two alignments in MAFFT [31]: one containing no missing loci across all individuals (complete) and another containing data for at least 75 % of taxa per locus (75 % complete), which returned alignments of 1114 loci and 4536 loci, respectively.

### Phylogenetic and biogeographic analyses

#### Sanger data

We first used Bayesian analyses with MrBayes 3.2.2 [32] of the ND2 and RAG-1 datasets independently in the context of the entire Agamidae to ensure that Draconinae was monophyletic. Once monophyly and lack of conflict between loci was established, we concatenated the two gene partitions for subsequent analyses. We used uniform priors in MrBayes 3.2.2 and partitioned the dataset by locus and codon within each locus for just the members Draconinae sub-family. We then assigned the GTR+ Γ substitution model for each partition and used three chains (two hot and one cold), and carried out 100 million generations, sampled every 10,000 generations. Due to the risk of substitution saturation, we performed analyses including and excluding the third codon position for the ND2 alignment. Convergence between chains, likelihood scores, and estimate sample size (ESS) values were evaluated using Tracer 1.6 [33] In order to obtain a reliable root age for divergence-time estimates within Draconinae, we expanded our ND2 and RAG-1 datasets to include data from all acrodont lineages. We analyzed this expanded dataset using eight acrodont fossils (Additional file 2: Table S2) within a Bayesian framework in BEAST 2.3 [34] using the fossilized-birth-death model [35, 36]. The fossilized-birth-death process provides a model for the distribution of speciation times, tree topology, and distribution of lineages sampled before the present, and treats the fossil observations as part of the prior on node time estimates. We used the root age for the Draconinae resulting from this analysis (85 MYA) as a minimum-age calibration for the root of the Draconinae for subsequent time of divergence estimates within the Draconinae clade.

#### Sequence-capture data

We performed likelihood analyses in RAxML v.8.1.20 [37] on concatenated datasets for the incomplete (4536 loci) and complete (1114 loci) matrices, using the GTR+ Γ substitution model, and ran 100 fast bootstrap replicates. In addition to the concatenated analysis, maximum likelihood gene trees were constructed for each of the UCE loci included in the complete matrix using Phyluce with RAxML v.8.1.20 [37], under default settings. Phyluce and RAxML were also used to generate gene trees for 500 multi-locus bootstraps [38]. Custom R-scripts (R v3.2.0; R Core Team 2015) and the R library Phybase [39] were then used to infer the STEAC [40] summary species tree for the original and bootstrapped data.

#### Grafted phylogeny and divergence dating

Using 85 MYA as a minimum age limit for the ancestor of the Draconinae, divergence dates for subclades were estimated in BEAST 2.3 using the ND2 and RAG-1 datasets with linked clock and tree models. We applied Birth-Death tree priors and constrained the relationships to match the results from the analyses of the UCE loci (blue nodes: Fig. 2b) and let the relationships within each species group be estimated by the BEAST analyses. We used a relaxed uncorrelated lognormal clock model and an exponential prior for the mean rate of each partition. Default values were used for all other priors, and the analysis was run for 150 million generations sampling every 12,000 generations, with chain stationarity, and ESS values were evaluated in Tracer 1.6. The first 25 % of trees were discarded as burn-in and the maximum clade credibility tree with median node heights was summarized using TreeAnnotator 2.3 [34]. We converted our alignments to fasta format using seqmagick (http://seqmagick.readthedocs.org/en/latest/). Then, with the estimate for divergence between *Mantheyus* and other draconine species of 85MYA, we estimated the TMRCA of subclades based on pairwise Hamming distances [41] between UCE loci (with a sequence saturation correction of 0.95) calculated through fastphylo [42], assuming a naïve strict clock. We carried out the calculations using a custom R-script [43]. Any loci where subgroup divergence times exceeded those of the calibration time were discarded due to the likelihood of incomplete lineage sorting and/or excessive rate variation. Using the same methods, we then estimated the time to most recent common ancestor (TMRCA) of the *Draco + Ptyctolaemus* and species group 1–4 clades using the estimated age of the Non-*Mantheyus* clade. The estimate of the TMRCA of species group 1–4 was then used to age the split between *Acanthosaura* and *Pseudocalates* (species group 1), and the ancestor of species groups 2/3/4. The species group 2/3/4 TMRCA estimate was then used to age the split between *Salea* and *Calotes* (species group 2 and 3), and the ancestor of species group 4. Finally, the estimate for the TMRCA of species group 4 was used to obtain an estimate of the TMRCA of *Certaophora/Lyriocephalus/Cophotis*.

Ancestral area reconstructions were performed using likelihood and Bayesian methods in LAGRANGE within the program RASP 3.0 [44], and in RevBayes 10.10 [45] respectively. Taxa were assigned to their biogeographic zone (Fig. 1) based on their modern day distributions and RevBayes reconstructions were visualized using the online resource Phylowood [46]. Traditionally, the Philippines is not classified as part of Sundaland however, we included taxa from this archipelago in the Sundaland biogeographic area because the entire Philippine agamid fauna is Sundaic in origin.

## Results

### Sanger mitochondrial and nuclear data phylogenetic analyses

The Bayesian analyses of the combined Sanger dataset recovered new relationships that have not been reported in any previous study (Fig. 2a). *Mantheyus* was recovered as sister to the remaining Draconinae. The next lineage to diverge was a well-supported clade containing *Draco,* and *Ptyctolaemus* (Fig. 2a). Lastly, there were four well-supported species groups (brown nodes: Fig. 2a). The relationships within each of these species groups were well supported. However, the relationships between the species groups were poorly resolved and characterized by short branches (pink nodes: Fig. 2a). As the resolution of the relationships between the species groups is vital for testing hypotheses of Indian or Eurasian origins, representatives of the taxa from each of these species groups were included in a phylogenetic reconstruction from analyses of UCE data.

#### Sequence-capture data phylogenetic analyses

There were 4536 loci with data for at least 75 % of the *n* = 23 individuals included in this study. These loci had an average length of 644.7 bp (S.D. = 249.7 bp), of which an average of 10.5 % of sites (S.D. = 20.0 %) were parsimony informative. The average amount of missing data per locus was 23.6 % (S.D. = 19.4 %), including both missing individuals (up to 25 % of individuals at each locus) and shorter sequence lengths for individuals that were present (Additional file 3: Table. S3). All analyses of the sequence-capture data were successful in resolving the problematic relationships recovered from the Sanger data (blue nodes; Fig. 2b) and recovered each of the four species groups within the Draconinae, with high support (brown nodes; Fig. 2b), consistent with the results from the Sanger datasets.

#### Biogeographic analyses, divergence dating, and ancestral areas

**Fig. 3:**
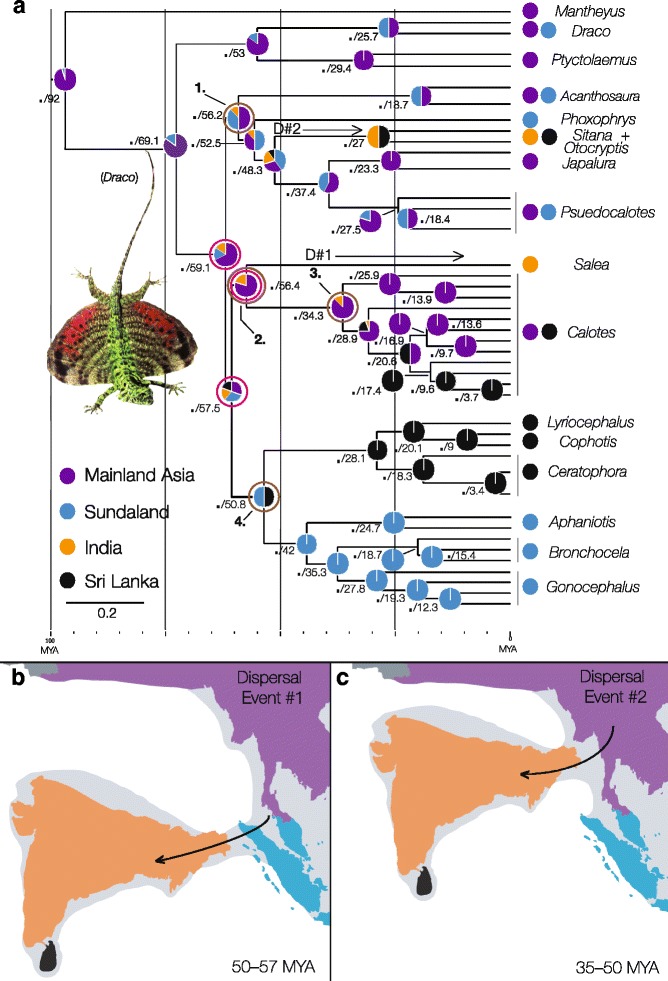
**a** Time-calibrated Bayesian analysis of ND2 and RAG-1 data, with black dots denoting nodes with posterior probabilities above 0.95, followed by the estimated divergence time for each node in MYA. Pink circles identify nodes where topology was constrained based on Likelihood and species tree analyses of UCE data (Fig. 2B). Brown circles indicate the four species groups. Biogeographic distributions of contemporary samples follow area coding depicted in Fig. 1, with probability of areas at ancestral nodes from our Bayesian analysis in RevBayes. Inferred dispersal events into India are labeled D#1 and D#2, resulting in Indian or Indian/Sri Lankan *Salea, Sitana,* and *Otocryptis*. **b** Hypothesized position of the ISC and an early Eocene land bridge allowing for the first inferred dispersal event (D#1 in **a**) from Eurasia into India, 50–55 MYA. **c**. Hypothesized position of the ISC and a middle-late Eocene land bridge allowing for the second first inferred dispersal event (D#2 in **a**) from Eurasia into India between 35–50 MYA (paleomaps modified from Klaus et al. [7])

**Fig. 4:**
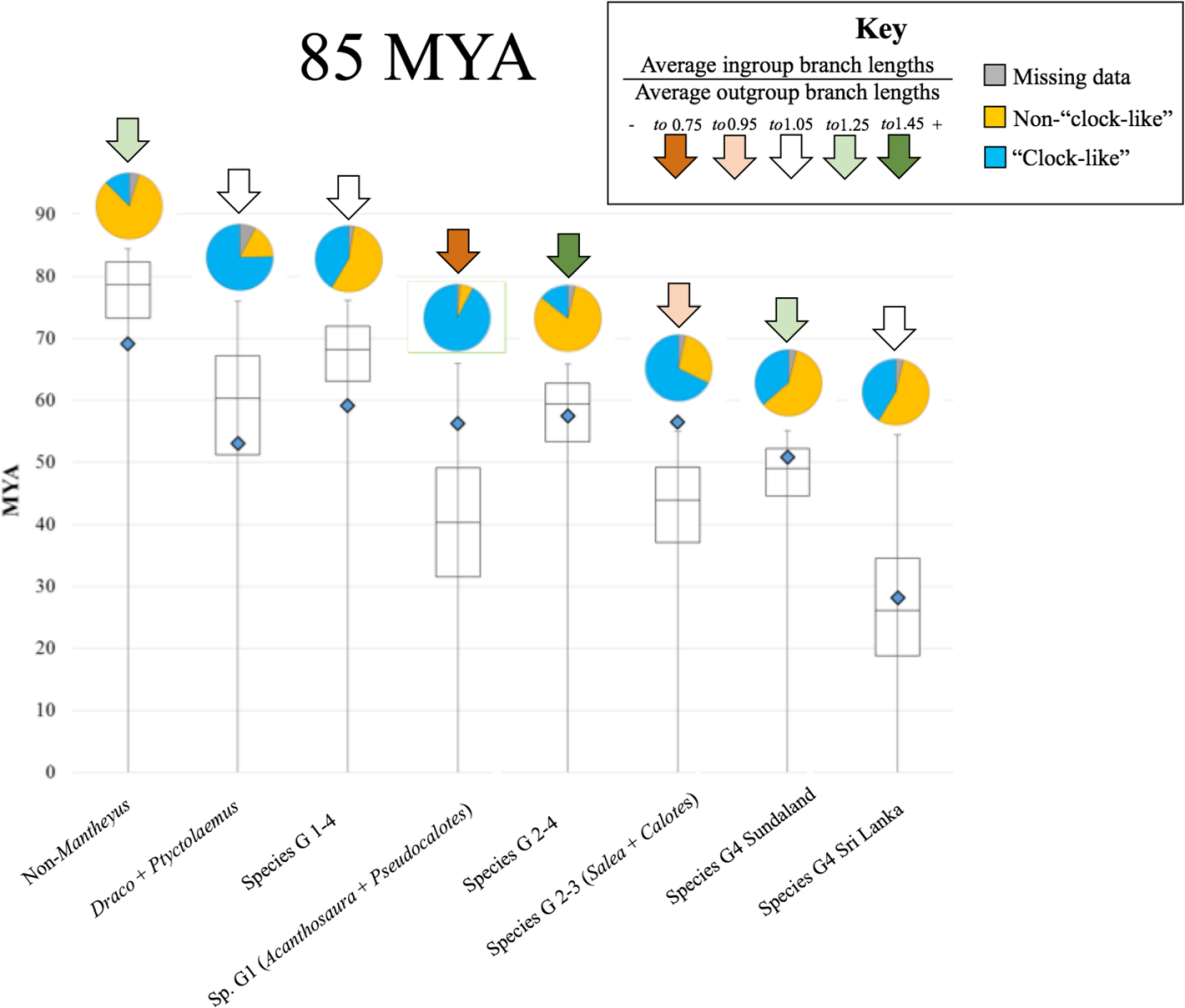
Box-and-whisker plots, showing results of our analysis using our UCE_divergence_timing R script (minimum, 25 % quartile, 75 % quartile, maximum) with a minimum estimate for the age of Draconinae of 85 MYA used to calibrate the ages of the Non-*Mantheyus* clade. For subsequent subgroups, the estimated age of the clades were contained within this calibration point. For each group’s divergence timing estimate, only loci that appeared “clock-like” (ingroup age estimate did not exceed the calibration age) were used. Percentages of loci that were “clock-like” versus non-“clock-like” (likely affected by rate variation or incomplete lineage sorting), and loci with missing data for outgroups (sister species of the groups of interest) are shown in pies above box-and-whisker plots (see key). Clades with red arrows show slow-downs relative to their outgroups i.e. average cumulative branch lengths leading to ingroup taxa from the ingroup/outgroup node are shorter than those leading to the outgroups (this appears to be correlated with underestimates of divergence times using the naïve strict clock method), clades with green arrows show rate speed-ups relative to their outgroups i.e. average cumulative branch lengths leading to ingroup taxa are longer than those leading to the outgroups. Bayesian estimates of divergences times performed in BEAST are shown as small blue diamonds, for comparison

Both of the methods employed to estimate ancestral ranges (LAGRANGE and RevBayes analyses) returned comparable estimates of ancestral areas, however, the RevBayes reconstructions were more conservative. Given the short branch lengths leading to some of the deeper nodes in our phylogeny, the RevBayes reconstructions are a better reflection of geology at the times of these nodes. Therefore only the RevBayes reconstructions are discussed. The grafted BEAST time-tree (Fig. 3a) was concordant with the phylogenies derived from the Sequence capture data and Sanger data (Fig. 2). The BEAST time-tree (Fig. 3) indicated the most recent common ancestor (MRCA) for the Draconinae originated approximately 92 MYA in mainland Asia ~30 million years after the ISC broke off Gondwanaland. The MRCA for *Draco* and its relatives most likely originated in mainland Asia 53 MYA and diverged from the other mainland Asian and Sundaic lineages around 69 MYA from a mainland Asian ancestor. The three remaining species groups appear to have diversified from one another rapidly between 51–59 MYA, most likely from a mainland Asia ancestor that existed approximately 59 MYA. The Indian endemic *Salea* (Species group 2) represents the first invasion of India (D#1: Fig. 3a), having diverged from a mainland Asian ancestor it shared with *Calotes* (Species group 3) approximately 56 MYA (Fig. 3a). The MRCA for *Acanthosaura* and *Psuedocalotes* (species group 1) was estimated at 56 MYA with a high probability that this ancestor originated in either mainland Asia or Sundaland (where both genera presently occur). Within this species group, we recovered support for a second invasion of India and Sri Lanka, with the ancestor of *Sitana* and *Otocryptis* originating from a predominantly Sundaic ancestor between 51–27 MYA (D#2: Fig. 3a). Lastly, the MRCA for the Sri Lankan and Sundaland radiations (species group 4) originated around 51 MYA in Sundaland or Sri Lanka (Fig. 3a). Within species Group 4, *Aphaniotis, Bronchocela,* and *Gonocephalus* appear to have diverged from one another 42 MYA and form the sister lineage to the Sri Lankan genera *Lyriocephalus, Cophotis,* and *Ceratophora* (Fig. 3a). The Sri Lankan lineages diverged from one another 28 MYA. We obtained these timing estimates for key divergences and dispersal events using Sanger data (as they were available for a broader taxonomic sample, including key fossils in comparison with the UCE data) in BEAST, with the topology constrained by the results from UCE data. We then crosschecked these estimates using the minimum divergence time for Draconinae of 85 MYA, and sequence divergence among UCE loci between clades of interest. This method is somewhat cruder than the BEAST estimates because it cannot account for among lineage rate variation. However, the estimates obtained using this approach were broadly comparable with results or our Bayesian analysis performed in BEAST (Fig. 4), offering support for our timing of key draconine dispersal events in Southeast Asia.

## Discussion

In this study, we utilized unprecedented sampling of the Draconinae, both in taxonomic diversity and genetic markers, to give fresh biogeographic insight into the origins of the Indian and Southeast Asian Draconinae lineages. In particular, the thousands of loci generated using sequence-capture and next-generation sequencing were successful in resolving previously problematic relationships within the Draconinae (brown nodes: Fig. 2). Using the fully resolved UCE phylogeny to constrain the topology of our Sanger dataset, we generated a grafted Bayesian time tree (Fig. 3a), which supported the hypothesis that there were at least two independent colonization events of India by Southeast Asian lineages during the Eocene. These results favor Moody’s [20] pre-collision hypothesis with the estimated times of the Eurasian invasions in accordance with the Eocene land bridges proposed by Acton, [13] and Ali and Aitchison [14]. These hypothesized land bridges would have connected areas of Eurasia (now Sundaland and the Thai-Malay peninsula) and the ISC before its collision, and are the likely conduits for terrestrial faunal exchange and range expansion in the lineages leading to today’s Indian subcontinent endemics *Salea, Sitana,* and *Otocryptis*.

### The Eocene exchange hypothesis

The first Draconinae invasion into India consisted of a lineage represented today by the endemic genus *Salea,* which descended from a mainland Asian ancestor that also gave rise to the Indochinese genus *Calotes*. This colonization event most likely resulted from an early Eocene land-bridge connection or an over-water dispersal event just prior to the ISC’s connection with Sundaland (Eurasia) 50–55 MYA (Fig. 3b). Given the sedentary and arboreal natural histories of extant draconine species, we feel the former hypothesis is more likely than the latter, although we acknowledge the possibility of both. We expect a broader sampling within this clade of Southeast Asian, and especially Indian, species will provide a better estimate of the ancestral area at this node (*Salea* + *Calotes*: Fig. 3a). The second dispersal event into India occurred with the divergence of the Indian and Sri Lankan endemics *Sitana* and *Otocryptis* from an ancestor most likely found in Sundaland during the middle Eocene. This colonization of the Indian subcontinent most likely was facilitated via a land bridge that connected the ISC with Sumatra and the Thai-Malay peninsula at 48 MYA. Additionally, the lineage sister to *Sitana* and *Otocryptis, Japalura,* and *Pseudocalotes,* is *Phoxophrys* (Fig. 3a). This genus is endemic to the lowland forests of Borneo and Sumatra—further supporting an India-Sundaland (Eurasia) connection via Sumatra and the southern portion of the Thai-Malay Peninsula during the middle Eocene. These independent colonization events not only support Moody’s [20] pre-collision biogeographic hypothesis, but also give additional phylogenetic support for Eocene land bridges postulated by Acton, [13] and Ali and Aitchison [14]. Our results contribute to a growing body of literature demonstrating the possibility of floral and faunal exchange between India and Eurasia during the Eocene, before the ISC’s hard collision 20–25 MYA (e.g. freshwater crabs: [7]; rhacophorid tree frogs: Li et al. [16]). Given the ecology of these organisms, and of the draconine species sampled here, we feel that it is less likely Eocene faunal exchanges occurred as the result of over water dispersal events. It is unclear whether the Eocene land bridges were two separate spatial/temporal features, versus possibly the same entity, just changing position as the ISC progressed northward. In either case, their existence may have provided continental connections between Southeast Asia and India during the Eocene, which could have allowed for terrestrial exchanges between these areas. These results collectively represent a broad-scale pattern of faunal exchange between the ISC and areas of Eurasia before its collision with Asia, at least partially facilitated by land bridges, which we term the “Eocene Exchange Hypothesis.” Furthermore, we believe the reoccurring and somewhat subjective disagreement between the Indian vs. Asian origins hypotheses [2–12, 16], have simply identified opposing perspectives of a broad geographic and temporal conduit of opportunity for faunal exchange between India and Eurasia. Future studies would benefit from an attempt to empirically focus on the timing and direction of faunal exchange between these biogeographic regions, rather than a prevalence of one scenario over the other.

### Revision of the age of draconinae

Our estimate for the age of Draconinae is significantly older than those previously published in broad scale squamate phylogenetic studies (most recently [47]). Our older estimates are largely due to our consideration of the acrodont fossils, *Mimeosaurus* and *Priscagama*, as leiolepids rather than stem agamids, following Estes et al. [48]. These fossils have had a rather turbulent history of classification, with various studies suggesting *Mimeosaurus* was allied with the Chameleonidae [49]; then hypothesized to be located along the branch leading to *Leiolepis* and *Uromystax* [20]; and lastly united with *Priscagama* in an extinct subfamily, Priscagaminae [50], considered to be a stem lineage of *Leiolepis* and *Uromystax* [51].

This confusion has persisted because when *Mimeosaurus* and *Priscagama* were first described, the contemporary genera *Leiolepis* and *Uromystax* were still included within the family Agamidae and demonstrated to be the sister group to the remaining agamids [20] (this relationship has been further confirmed with molecular data [21, 23, 52, 53]. However, Estes et al. [48] removed *Leiolepis* and *Uromystax* from the Agamidae and placed them in their own family (the Leiolepidae), and this taxonomy has not been followed by subsequent studies. Thus, the acrodont fossils of *Priscagama* and *Mimeosaurus* have been consistently considered as stem fossils for all agamids and not their sister group, *Leiolepis* and *Uromystax*. We followed the taxonomy of Estes et al. [48] and considered *Mimeosaurus* and *Priscagama* as stem leiolepids and not stem agamids. It was this placement that lead to our older estimates of Draconinae origins (85–92 MYA). However, this estimate is consistent with the ages of new amber agamid fossils being described out of Indochina and previous studies on Iguanian lizards ([54]; Bauer et al., unpublished data; *personal communication with JLG and PW*). We recommend that researchers continue to follow the taxonomy of [48] with the recognition of the Leiolepidae as a distinct family and the placement of priscagamine fossils as stem to *Leiolepis* and *Uromystax*, as suggested in the original descriptions of these fossils [20, 50, 51].

## Conclusions

The use of additional taxa, sequence-capture data, and newer geological models—all data not available to previous studies on Draconinae—resulted in novel and well-resolved relationships, leading to new biogeographic insights in this unique subfamily of lizards. Using these biogeographic insights and a broad comparison with previous biogeographic literature, we propose the Eocene Exchange Hypothesis, and the simple but well supported assumption that land bridges may have facilitated a broad-scale pattern of faunal exchange between the ISC and areas of Eurasia before its collision with Asia during the Eocene. We expect that with additional sampling of Indian and mainland Asian species, some factors that may have biased our biogeographic interpretations within the Draconinae to (i.e., Indian extinction events), can be evaluated. In addition, sampling of additional draconine species will allow us to test more fine-scaled hypotheses concerning dispersal and diversification within this group. Our phylogenomic analysis add to a growing body of knowledge addressing the effects of the ISC’s collision on biogeography and offers new ideas to be tested by future studies.

## Additional files

Additional file 1: Table S1. List of all the species and their associated localities used in this study. (XLSX 56 kb) Additional file 2: Table S2. List of all the fossil calibrations, their ages, and their associated references, used in this study. (DOCX 72 kb) Additional file 3: Table S3. Per locus metrics for the UCE sequence capture data. (XLSX 289 kb)

## Abbreviations

ISC: Indian subcontinent
EEH: Eocene Exchange Hypothesis
MYA: million years ago
