# Supplementary material for: The Eurasian invasion: phylogenomic data reveal multiple Southeast Asian origins for Indian Dragon Lizards
Source: BMC Evol Biol. 2016 Feb 19;16:43. doi: 10.1186/s12862-016-0611-6 (PMC4759850; doi:10.1186/s12862-016-0611-6)
Supplement: Additional file 2: Table S2. — List of all the fossil calibrations, their ages, and their associated references, used in this study. (DOCX 72 kb) [file 12862_2016_611_MOESM2_ESM.docx]

| **Acrodont Fossil** | **Age** | **Placement and Citation** |
| --- | --- | --- |
|  |  |  |
| Fossil chameleonid with morphological similarities to *Rhampholeon* | 18  MYA | Stem to *Rhampholeon* (Rieppel et al. 1992). |
| *Mimeosaurus* and *Pricagama* (Priscagamidae) | 72–80 MYA | Stem to *Leiolepis and Uromastyx* (Gilmore 1943; Moody 1980; Gao, K., and M. A. Norell. 2000; Gradstein et al. 2012). |
| *Leiolepidinae* | 53 MYA | Earliest stem for *Leiolepis* (Wing et al. 2000; Smith 2011; Smith and Gauthier 2013). |
| *Uromastycinae* | 48 MYA | Earliest stem for *Uromastyx* (Averianov and Danilov 1996; Gradstein et al. 2012). |
| *Paleochameleo* | 39 MYA | Reevaluated to be a stem lineage for *Uromastyx* (Moody 1980). |
| *Barbatus* | 37 MYA | Stem for *Uromastyx* (Head et al. 2013) |
| *Physignathus* | 19 MYA | Earliest possible stem for the genus (Covacevich et al. (1990) |
| *Phrynocephalus* | 5 MYA | Earliest possible stem for the genus (Zerova and Chkhikvadze, 1984). |
